# The Role of Physical Fitness in Cognitive-Related Biomarkers in Persons at Genetic Risk of Familial Alzheimer’s Disease

**DOI:** 10.3390/jcm8101639

**Published:** 2019-10-07

**Authors:** Chia-Liang Tsai, H.-Sunny Sun, Yu-Min Kuo, Ming-Chyi Pai

**Affiliations:** 1 Institute of Physical Education, Health and Leisure Studies, National Cheng Kung University, Tainan 701, Taiwan; 2 Institute of Molecular Medicine, College of Medicine, National Cheng Kung University, Tainan 701, Taiwan; 3 Department of Cell Biology and Anatomy, College of Medicine, National Cheng Kung University, Tainan 701, Taiwan; 4 Division of Behavioral Neurology, Department of Neurology, National Cheng Kung University Hospital, College of Medicine, National Cheng Kung University, Tainan 704, Taiwan; 5 Alzheimer’s Disease Research Center, National Cheng Kung University Hospital, Tainan 704, Taiwan

**Keywords:** Alzheimer’s disease of family history, neural oscillation, inflammatory cytokines, amyloid-β, BDNF, cognition, physical fitness, APOE-4

## Abstract

**Introduction:** Nondemented people with a family history of Alzheimer’s disease (ADFH) and the ApoE-4 allele have been demonstrated to show a trend for a higher probability of cognitive decline and aberrant levels of cognitive-related biomarkers. However, the potential interactive effects on physical fitness have not been investigated. **Purpose:** The primary purpose of this study was to determine whether ADFH individuals with the ApoE-4 genotype show deviant brain event-related neural oscillatory performance and cognitively-related molecular indices. A secondary purpose was to examine the interactive effects on physical fitness. **Methods:** Blood samples were provided from 110 individuals with ADFH to assess molecular biomarkers and the ApoE genotype for the purpose of dividing them into an ApoE-4 group (*n* = 16) and a non-ApoE-4 group (*n* = 16) in order for them to complete a visuospatial working memory task while simultaneously recording electroencephalographic signals. They also performed a senior functional physical fitness (SFPF) test. **Results:** While performing the cognitive task, the ApoE-4 relative to non-ApoE-4 group showed worse accuracy rates (ARs) and brain neural oscillatory performance. There were no significant between-group differences with regard to any molecular biomarkers (e.g., IL-1β, IL-6, IL-8, BDNF, Aβ1-40, Aβ1-42). VO_2max_ was significantly correlated with the neuropsychological performance (i.e., ARs and RTs) in the 2-item and 4-item conditions in the ApoE-4 group and across the two groups. However, the electroencephalogram (EEG) oscillations during visuospatial working memory processing in the two conditions were not correlated with any SFPF scores or cardiorespiratory tests in the two groups. **Conclusions:** ADFH individuals with the ApoE-4 genotype only showed deviant neuropsychological (e.g., ARs) and neural oscillatory performance when performing the cognitive task with a higher visuospatial working memory load. Cardiorespiratory fitness potentially played an important role in neuropsychological impairment in this group.

## 1. Introduction

Alzheimer’s disease (AD) is a common progressive neurodegenerative disorder characterized by neurocognitive decline that can precede the diagnosis of AD dementia by at least 2 to 3 years [1,2,3]. A greater understanding of preclinical biomarker changes that occur in AD might facilitate earlier identification of the disease. To date, no curative pharmacotherapy exists for AD. Therefore, identification of the earliest stage of preclinical AD disease in order to arrive at effective treatments is both urgent and important.

The apolipoprotein E (ApoE) allele is the major known genetic risk factor for AD [4]. However, the ApoE genotype alone is not considered an effective predictor in nondemented persons. For example, only the ApoE-4 allele on chromosome 19 is associated with cognitive decline over time, lowered onset age, and increased risk for late-onset sporadic AD in nondemented older adults [5], whereas ApoE-2 may confer protection [6]. ApoE-4 is associated with declining memory [7], impaired neuronal plasticity [8,9], and altered synaptic morphology [10,11]. In comparison to noncarriers, cognitively normal ApoE-4 carriers show differences in functional activation [12], glucose metabolism [13], brain structure [14], and anisotropic diffusion [15]. Nondemented elderly adults with ApoE-4 heterozygotes or homozygotes have been demonstrated to show the risk of developing impaired cognitive function 3–6 years in the future, as assessed using the Mini-Mental Status Examination (MMSE) [16,17]. Thus, ApoE-4 is related to amnestic mild cognitive impairment (MCI) and is one of the most effective predictors of clinical progression from MCI to AD dementia [18].

A family history of Alzheimer’s disease (ADFH) in one of the most important risk factors established for the disease [19]. Previous studies have demonstrated that a parental family history of AD is associated with altered brain microstructure manifested as lower fractional anisotropy (FA) in regions of the brain known to be affected by AD [20]. Middled-aged AD siblings and children were found to be more likely to show a decline in memory and in selected intelligence subtests across a 4-year follow-up interval when compared to controls [21,22].

ADFH is the most important risk factor established for the early-onset disease, especially with pedigrees with an autosomal dominant pattern of inheritence [19]. Previous studies have demonstrated that nondemented middle-aged ADFH individuals with ApoE-4 carriers relative to noncarriers show reduced beneficial practice effects on memory tests and an accelerated decline in recall and verbal list learning over an average follow-up interval of 33 months [23]. Cognitively healthy middle-aged and older adults who are almost first-degree AD relatives have also been reported to show subclinical cognitive problems in recall, working memory, attention, and verbal learning among ApoE-4 carriers [24,25,26]. Among the heterogeneous MCI population, individuals at higher risk of AD (i.e., with ApoE-4 alleles) have been found to exhibit poorer neuropsychological performance in spatial navigation [27]. In addition, compared to individuals with ADFH who exhibit lower FA values, an additive effect of family history and ApoE-4 was found in which individuals with ADFH and ApoE-4 allele had the lowest FA values.

With the exception of a known genetic marker, discovery of additional factors for individuals with ADFH may help in early detection research to identify who will be more likely to develop AD. Physical fitness may influence individual susceptibility in the ApoE-4 allele and increased risk of cognitive decline with advancing age. Head et al. (2012) found that in cognitively normal individuals with the ApoE-ε4 allele, regular exercise at levels recommended by the American Heart Association modulates amyloid deposition in cerebrospinal fluid (CSF) and mean cortical binding potential (MCBP), with a greater exercise effect on MCBP values in ε4-positive individuals compared to ε4-negative individuals and augmented risk for amyloid deposition in ε4-positive individuals leading a sedentary lifestyle [28]. Etnier et al. (2007) demonstrated that aerobic fitness is a potential factor that may modulate ApoE-4 and cognitive functions involving memory in individuals at most genetic risk for Alzheimer’s disease [29].

AD is characterized not only by brain synaptic and neuronal loss but also by extracellular accumulation of amyloid-β (Aβ) and inflammation [30,31,32]. Aβ accumulation initiates neurodegeneration and is associated with Alzheimer’s type clinical dementia [33,34]. ApoE-4 increases Aβ aggregation and impairs soluble Aβ clearance in the brain relative to other isoforms of expressed human ApoE [35,36]. In the amnestic MCI group, Aβ positive relative to Aβ negative patients exhibited higher frequency of ApoE-4 and poorer neuropsychological performance (e.g., spatial recognition and working memory) [37]. Higher levels of pro-inflammatory cytokines have been suggested to be independent, strong predictors of increased risk of progression to AD dementia [38,39], with higher serum levels of interleukin (IL)-1β, IL-6, and IL-8 being reported in patients with MCI or AD dementia [40,41]. Reduced brain-derived neurotrophic factor (BDNF) levels are considered to be a pathogenic event in early AD and even in the earliest, prodromal stages of dementia (e.g., MCI) [42,43]. Individuals with ApoE-4 carriers have been demonstrated to show lower BDNF levels than non-ApoE-4 carriers in females with AD, but not in males with AD [44]. The levels of Aβ, BDNF, and pro-inflammatory cytokines have been found to be strongly associated with physical exercise levels and cognitive functions [45,46,47,48].

Some indices that have been useful in predicting who will develop dementia include preclinical levels of neurophysiological and biochemical markers. Nondemented ADFH individuals with ApoE-4 carriers have been reported to show neuropsychological problems on different cognitive tests [23,24,25,26]. Thus far, no study has examined the potential neurophysiological mechanisms underlying visuospatial working memory impairment in this group. Electroencephalogram (EEG) signals recorded from the scalp can be decomposed into different oscillatory frequency components (e.g., theta, alpha, and beta frequencies) through a wavelet analysis, which represents several aspects of cognitive processing (e.g., attention, working memory, and long-term memory) [49], and can be simultaneously inspected as a function of time and frequency [50] to reflect neural mechanisms associated with cortical information processing and brain dysfunction [51,52,53]. Event-related neural oscillation was thus to explore the neurophysiological performance in the present study. In addition, as mentioned above, individuals with MCI/AD/ApoE-4 exhibit aberrant cognitive-related molecular markers that have not been explored in those with ADFH and the ApoE-4 genotype.

To sum up, the primary purpose of this study was to explore the levels of AD- and cognition-related biochemical markers and the performance on electroencephalogram (EEG) oscillations in ADFH individuals with the ApoE-4 genotype when performing a visuospatial working memory task. Furthermore, physical fitness level is strongly associated with the executive function and AD-related molecular markers. The interactive effects on physical fitness levels and neurocognitive performance/biochemical indices in individuals with ADFH and ApoE-4 were also explored to provide the foundations for clinical references on AD prevention. We believe that the present study can provide additional insights into the role of physical fitness in cognitive-related biomarkers in persons at genetic risk of familial Alzheimer’s disease and a potential clinical application for the prevention of AD.

## 2. Materials and Methods

### 2.1. Participants

One hundred and ten participants, aged 30–70 years, were included with a family history of AD (ADFH), defined as first-degree relatives (siblings and children) with a clinical diagnosis of AD to reduce the interacting effects of genetic and environmental factors. DNA was obtained from their blood samples to determine the ApoE genotypes. ADFH individuals were subsequently divided into 2 ApoE genotype groups, ɛ4 carriers (*n* = 16, all ɛ3/ɛ4 heterozygotes) and non-ɛ4 carriers (*n* = 94, 75 ɛ3/ɛ3 homozygotes and 19 ɛ2/ɛ3 heterozygotes). The APOE-4 group comprised ADFH individuals with ɛ4 carriers. In order to reduce the influence of trial number or sample size on EEG oscillation measures [54], the non-APOE-4 group was randomly selected from the non-ɛ4 carrier group and comprised 16 ADFH individuals with ɛ3/ɛ3 homozygotes (*n* = 14) or ɛ2/ɛ3 heterozygotes (*n* = 2). All participants had no other neurological, medical, or psychiatric illnesses (e.g., depression) that could affect memory or cognitive processing, significant cerebrovascular disease, musculoskeletal impairment, nor were they using antidementia medicine. All participants were right-handed, as assessed by the Edinburgh Handedness Inventory, and had normal (or corrected to normal) vision based on the minimal 20/20 standard. Written informed consent was obtained in accordance with the procedures set by the local Institution Ethics Committee.

### 2.2. Procedure

All participants were required to refrain from strenuous exercise for at least 24 h and were asked to avoid food, caffeine, smoking, and alcohol intake for at least 12 h. Each participant attended the cognitive neurophysiology laboratory for one session at about 8:30–9:30, which included the attainment of an informed consent form, blood withdrawal, the completion of a demographic and medical history questionnaire, the Mini-Mental State Examination (MMSE), the Montreal Cognitive Assessment (MoCA), Addenbrooke’s Cognitive Examination-III (ACE-III), the Beck depression inventory-II (BDI-II), a social participation assessment, and a handedness inventory, where the cognitive task test was administered while simultaneously recording the electroencephalographic signals. Then, two certified personal trainers completed all senior functional physical fitness (SFPF) assessments [55] and estimated VO_2max_ using the Rockport Fitness Walking Test [56], in which each participant was required to walk one mile as quickly as possible, with their heart rate (HR) being continuously recorded using a Polar heart rate (HR) monitor (RX800CX, Polar Electro Oy, Kempele, Finland).

### 2.3. Cognitive Task

Since deficits in spatial navigation [27] and short-term memory [57] may be early markers in Alzheimer’s disease-related individuals (e.g., familial Alzheimer’s disease or amnestic mild cognitive impairment) with or without the ApoE-4 allele, a modified visuospatial working memory task was adopted in the present study [58].

**Figure 1:**
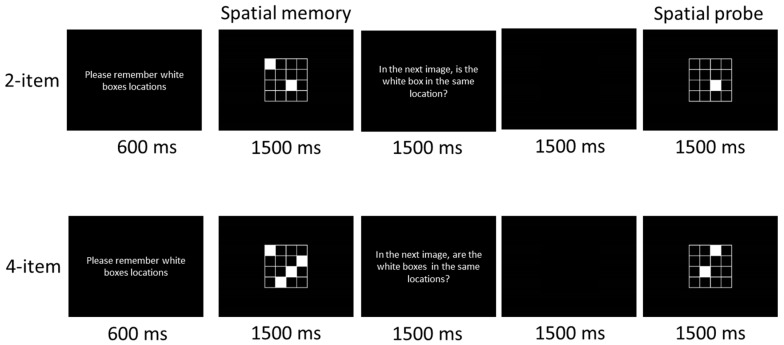
Schematic of the visuospatial working memory task.

As illustrated in Figure 1, a modified version of a visuospatial working memory task [59] was adopted in the present study. In the task, each trial began with the white demand statement, “Please remember the location of the white boxes,” which was placed at eye level with a viewing distance of approximately 100 cm, with a 600 ms duration. The statement appeared in the center of a computer screen (width = 43 cm) with a black background after a 100 ms warning tone (1000 Hz, 75 db SPL) presented binaurally through headphones. Then, a fixed rectangular area with white outlines of 9 squares appeared 1500 ms after the offset of the demand statement. Two or four of the squares (i.e., 2-item or 4-item condition, respectively) were filled with white, and their locations were randomly chosen in each trial. Following a 1500 ms exposure to this “spatial memory” stimulus, the computer monitor was turned to a black background for 3 s, during which the question, “In the next image, is/are the white box (es) in the same location(s)?” appeared for 1500 ms prior to a 1500 ms black background. Then, the “spatial probe” stimulus included the same 9 squares but with only 1 (2-item condition) or 2 (4-item condition) where the 9 squares were filled. In half of the trials, the location(s) of the box (es) matched the either (or 2) of location(s) of the filled 2 or 4 squares presented in the previous “spatial memory” stimulus. Matching and nonmatching trials were presented in a random order. The participants were asked to respond as quickly and accurately as possible by pressing the “M” on the computer keyboard with the index finger of the right hand (“yes”) or the “Z” with the index finger of the left hand (“no”). Individual trials were separated by a 3.5 s inter-trial period. Each participant completed a total of 130 trials (50 2-item trials and 80 4-item trials). The experimental session was divided into three blocks (one for the 2-item condition and two for the 4-item condition), with a 3-min break after each block of trials.

### 2.4. Blood Sampaling and Analysis

A 10 mL blood sample was taken from the antecubital vein for an analysis of serum inflammatory cytokine (e.g., IL-1β, IL-6, and IL-8), BDNF, Aβ1-40, and Aβ1-42 levels. To permit clotting, the blood samples were incubated at room temperature (BD Vacutainer Plus), after which they were centrifuged at 2500 rpm at 4 °C for 15 min (Hettich Mikro 22R, C1110, Hettich, Tuttlingen, Germany). The serum was stored at −80 °C in small aliquots. Inflammatory cytokines and BDNF levels were analyzed using human cytokine antibody-immobilized magnetic beads (Millipore, Billerica, MA, USA), and a Luminex 200 analyzer (Luminex, Austin, TX, USA) was used to perform the measurements. The levels of Aβ1-40 and Aβ1-42 biomarkers were measured using single molecule counting (SMC^®^) immunoassay technology with commercially available kits obtained from Millipore and Sigma (Aβ1-40: # 03-0145-00, Aβ1-42: # 03-0146-00). A single individual performed the entire procedure used to determine the molecular markers in order to avoid interoperator bias. In terms of inter- and intra-assay precision, blood samples were run on multiple plates over a period of 3 days. Spiked and unspiked samples were within 20% across experiments.

### 2.5. Time-Frequency Analysis

SPM8 for MEG/EEG (Welcome Department of Cognitive Neurology, London, UK; www.fil.ion.ucl.ac.uk/spm/) and custom MATLAB (MathWorks) scripts [60] were used to conduct this analysis. Prior to conducting the time–frequency analysis, large artifacts in the continuous EEG data were identified, and the maximum eye-blink was set. Eye-blink peaks were derived from vertical electrooculographic activity using both a regression and correlations, and a correction for the eye-blinks was applied to the EEG data [61]. These data were used to perform a correction for all channels. The offline ocular-corrected EEG data were locked to the stimulus onset and were segmented into epochs set at −1500 to 1500 ms relative to the stimulus onset. Trials containing artifacts exceeding ±150 uV were discarded [62]. A continuous Morlet wavelet transform (Morlet wavelet factor = 6) for single-trial data at a frequency band ranging from 2 to 65 Hz [63] was used to compute the power estimates. The oscillatory power, which herein was defined as the square of the modulus of the resulting complex number, was subsequently averaged across trials. The averaged oscillatory power for each condition for each participant was rescaled based on the baseline value, which ranged from −300 to −100 ms, before the stimulus onset [60] after taking the log10 transform of this quotient. All trials were averaged for each condition. The Fz electrode was selected for further analysis since oscillatory alpha activity at this site has been associated with a fitness effect on executive functioning [64], Further, through functional magnetic resonance imaging, the prefrontal cortex was activated when the individuals performed the visuospatial working memory task adopted in the present study [58]. For each time sample and frequency, these log-transformed increases in signal power relative to the baseline were used as the measure of interest for the statistical analysis that followed. To test the *trial type* effect (2-items, 4-items; paired *t*-tests) and *group* effect (APOE-4, non-APOE-4; independent *t*-tests), a *q* < 0.05 with a false discovery rate (FDR) corrected [65] or *p* < 0.01 uncorrected for multiple comparisons was employed.

### 2.6. Statistical Analysis

The demographic characteristics and the differences in biochemical markers between the obesity and healthy-weight groups were compared using the Student’s unpaired t-test. In order to evaluate the neuropsychological performance, the participants’ reaction times (RT) and accuracy rates (AR, percentage correct) were separately analyzed using a mixed design, factorial, and repeated-measures analysis of variance (RM ANOVA). The *Group* (ApoE-4 vs. non-ApoE-4) was the between-subjects factor, and the *Condition* (2-item vs. 4-item) served as the within-subject factor. The ARs and mean RTs of those trials that were accepted served as the dependent variables. The normality and homogeneity of variance assumptions were respectively confirmed by the Kolmogorov–Smirnov test and the Lilliefors and Levene’s tests. In cases where the RM ANOVAs exhibited statistically significant main effects due to the factors and their interactions, posterior comparisons of the mean values were carried out with multiple pairwise comparisons (adjusted using the Bonferroni correction). If deemed appropriate, the Greenhouse–Geisser (G–G) procedure was applied to correct the degrees of freedom whenever a major violation of the sphericity assumption was detected in the RM ANOVA, with more than two degrees of freedom. To complement the use of significance testing, the effect size (i.e., partial η2: *η_p_*^2^) was additionally reported, where the magnitude of effects was based on the following standards: 0.01–0.059 represented a small effect, 0.06 to 0.139 represented a medium effect, and >0.14 represented a large effect [66]. In addition, a further across-group investigation was conducted using a Pearson’s r product-moment correlation coefficient to determine the associations between the physical fitness scores and neurocognitive performances/the levels of molecular indices (if between-group significance was found), where *p* < 0.05 was deemed statistically significant.

## 3. Results

### 3.1. Participant Characteristics

As shown in Table 1, the ApoE-4 and non-ApoE-4 groups were matched at the group level on sociodemographic variables (e.g., age, years of education, systolic and diastolic pressure, height, weight, BMI, and social participation) (all *p* > 0.05). A chi-square analysis failed to reveal any significant between-group gender distribution differences. The cognitive ability (e.g., MMSE, MoCA, and ACE-III), depressive state (e.g., BDI-II), and SFPF scores also revealed nonsignificant between-group differences.

### 3.2. Behavioral Performance

#### 3.2.1. Accuracy Rates (ARs)

The RM ANOVA on the ARs revealed a significant main effect of *Condition* (*F*(1, 30) = 91.02, *p* < 0.001, *η_p_*^2^ = 0.75). The post hoc analyses indicated that the ARs for the 2-item condition (91.8%) were larger than those for the 4-item one (71.6%) for both groups. These main effects were superseded by the *Group* × *Condition* (*F*(1, 30) = 11.01, *p* = 0.002, *η_p_*^2^ = 0.27) interaction. Post hoc analyses indicated that the ApoE-4 group was only worse in the 4-item condition as compared to the non-ApoE-4 group (66.00 ± 17.17% vs. 77.19 ± 10.65%, *p* = 0.034).

#### 3.2.2. Reaction Time

The RM ANOVA on the response times (RTs) only revealed a significant main effect of *Condition* (*F*(1, 30) = 92.40, *p* < 0.001, *η_p_*^2^ = 0.76). The post hoc analyses indicated that the two groups had slower RTs for the 4-item condition (884.6 ms) than the 2-item condition (1047.0 ms).

### 3.3. Alpha Power Oscillations

**Figure 2:**
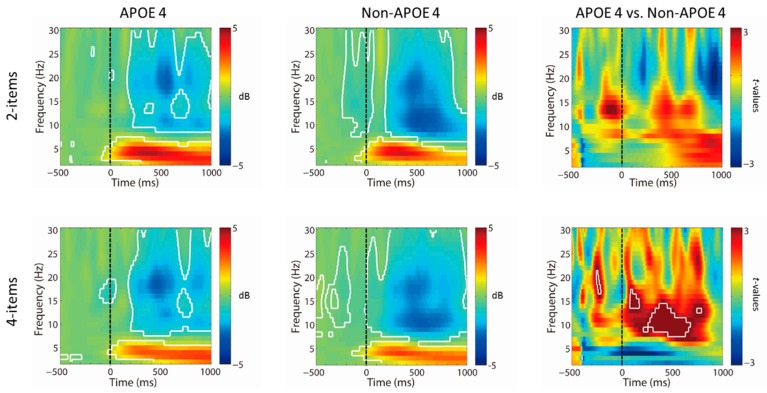
Time–frequency decomposition with Morlet wavelets in the frontal region (Fz). Stimulus-locked rescaled oscillatory power averaged across midfrontal region stimulus in ApoE-4 and non-ApoE-4 groups. The results revealed that the non-ApoE-4 group showed significantly greater decreases in electroencephalogram (EEG) power at the alpha band (9–13 Hz) in the 4-item condition relative to the ApoE-4 group at approximately 250–550 ms following target onset (all *p* < 0.01, uncorrected).

As illustrated in Figure 2, in terms of spectral EEG power, both trial conditions exhibited similar increases in theta (4–7 Hz) (all *q* < 0.05, FDR corrected) and decreases in alpha (9–13 Hz) (all *q* < 0.05, FDR corrected) and beta (15–30 Hz) (all *q*< 0.05, FDR corrected) oscillatory power after stimulus onset (see Figure 2), relative to the baseline period. No differences in conditions in the event-related power changes across all time points and frequencies (all *q* > 0.05, FDR corrected) were observed. However, the two-sample *t* tests revealed that the non-ApoE-4 group showed significantly greater decreases in oscillatory power at the alpha band (9–13 Hz) only in the 4-item condition relative to the ApoE-4 group approximately 300–550ms following target onset (all *q* < 0.05, FDR corrected), whereas this effect was not found in the 2-item condition.

### 3.4. Molecular Biomarkers

As shown in Table 2, there were no significant differences (all *p* > 0.05) at the group level with regard to any molecular biomarkers (e.g., IL-1β, IL-6, IL-8, BDNF, Aβ1-40, Aβ1-42) in the ApoE-4 and non-ApoE-4 groups.

### 3.5. Correlation

As shown in Table 3, among all the scores for the SFPF and cardiorespiratory tests, the VO_2max_ showed significant correlations with ARs and RTs in the 2-item and 4-item conditions in the ApoE-4 group. For the non-ApoE-4 group, chair sit-and-reach scores and VO_2max_ were only significantly correlated with the AR in the 2-item condition. Across the two groups, there were also significant correlations between VO_2max_ and ARs or RTs in the 2-item and 4-item conditions. In addition, the scores for the grip and arm curl showed significant correlations with the ARs in the 2-item condition. The grip score was also significantly correlated with the AR in the 4-item condition.

In order to determine whether the group differences in EEG oscillations could be explained by physical fitness performance, the EEG oscillations during visuospatial working memory processing for the two conditions were correlated with the SFPF scores and the cardiorespiratory tests. However, none of these correlations reached the significance level for either group (all *q* > 0.05, FDR corrected) as well as across all participants (all *q* > 0.05, FDR corrected).

## 4. Discussion

The current study investigated the levels of AD- and cognition-related molecular biomarkers and the neurophysiological mechanisms of visuospatial working memory impairment in ADFH individuals with ApoE-4 carriers to extend the body of research exploring the neuropsychological performance in such a group [24,25,26]. To provide a clinical reference for AD prevention, the interactive effects on physical fitness levels and neurocognitive performance/biochemical indices in ADFH individuals with ApoE-4 carriers were also examined. We found that although the ApoE-4 group showed comparable scores on senior functional fitness tests, RTs, and oscillatory responses in the theta and beta frequency range when performing the visuospatial working memory task, they exhibited worse ARs and deviant alpha oscillations in the 4-item condition. However, there were no significant differences at the group level with regard to any molecular biomarkers (e.g., IL-1β, IL-6, IL-8, BDNF, Aβ1-40, Aβ1-42) between the ApoE-4 and non-ApoE-4 groups. In the ApoE-4 group, cardiorespiratory fitness scores showed significant correlations with neuropsychological performance in the two low and high working-memory load conditions, suggesting a potential clinical application for the prevention of AD.

Oscillatory responses in the theta and alpha frequency range are associated with the engagement of working memory processes and play a fundamental role in visuospatial working memory [53], with theta oscillation being related specifically to working memory processes (e.g., memory encoding and retrieval) and the functioning of central executive [67,68], and alpha oscillation to attention and memory processes, especially in response to increased memory load and demands [51,69,70]. Accumulating evidence indicates that the ApoE-4 allele is related to impaired neuronal plasticity [8,9] and altered synaptic morphology [10,11]. A study of middle-aged nonsymptomatic ADFH individuals with ApoE-4 carriers revealed abnormal prolongation of P300 latency when performing an auditory oddball paradigm as compared with ADFH individuals without ApoE-4 carriers [71]. In terms of neural oscillations, Sun et al. (2017) demonstrated that significant abnormalities in hippocampal synaptic function occur in as little as 4 months in ApoE-4 mice, which showed impaired carbachol-induced hippocampal theta oscillations without changes in synaptic vesicle recycling compared to ApoE-3 mice [72]. Further, Babiloni et al. (2004) found that the amplitude of EEG alpha sources in temporal, occipital, and limbic areas was lower in ApoE-4 carriers than in noncarriers in both MCI and AD participants when cortical sources are estimated from resting eyes-closed EEG rhythms [73]. However, the present study only partly supported these previous findings. That is, only the performance of the alpha but not the theta oscillation was significantly different between the middle-aged and older ApoE-4 and non-ApoE-4 groups. Since frontal midline theta oscillations are related to the central executive functions of working memory [68], the ApoE-4 group showed comparable oscillatory theta activity to that of the non-ApoE-4 group, suggesting that ADFH individuals with ApoE-4 carriers still process new information efficiently when they are required to dynamically integrate spatial information into decision-making processes. However, working memory can be decomposed into a central executive component and short-term memory, which are associated with the controlled attention processes necessary for retaining information in spite of interference or distraction [74]. In the present study, the ApoE-4 relative to the non-ApoE-4 group exhibited deviant alpha oscillations, suggesting that cognitive functioning related to attention and memory processes in terms of information maintenance was impaired in the ADFH individuals with ApoE-4 carriers when performing the visuospatial working memory task, especially when exposed to increased memory load and demands [51,69,70]. It is worth noting that the non-ApoE-4 group only showed significantly greater decreases in alpha oscillation and better ARs in the 4-item condition as compared to the ApoE-4 group. This finding implies that middle-aged and elderly ADFH individuals with ApoE-4 carriers still have sufficient cognitive ability to perform lower working-memory load tasks before progression to MCI or AD dementia.

The present study provides insight into how an early deviant alpha underlying ApoE-4 in ADFH individuals affects visuospatial working memory performance. In fact, event-related desynchronization in the alpha band specifically reflects an increased excitability level of neurons in the involved cortical areas, which could be related to enhanced information transfer in thalamocortical communications [51]. Cantero et al. (2009) found that the level of functional dependence between corticocortical EEG sources and thalamic and cortical sources involved in the generation of lower alpha oscillations is abnormally facilitated in individuals with MCI compared to healthy elderly controls [75]. Since individuals with ApoE-4 carriers are at increased risk of developing MCI and AD [76], abnormalities in EEG-alpha oscillations elicited by the working-memory-related task could thus be considered a promising biomarker of MCI or early-onset AD.

Importantly, the ApoE-4 allele has a dose-related effect on increasing risk and lowering onset age for the late onset familial and sporadic forms of AD [5]. In the present study, after assessing the ApoE genotype, ADFH individuals with ApoE-4 carriers were all heterozygotes (ɛ3/ɛ4 genotype). Although a previous study reported that in the families with late onset AD, homozygosity for ApoE-4 is virtually sufficient to cause AD by age 80 [5], a single copy of ApoE-4 also confers a 20% risk of developing the disease [76]. In addition, Small et al. (2000) demonstrated that a single copy of the ApoE-4 allele is also associated with lower cerebral cortical metabolism (e.g., the parietal, temporal, and posterior cingulate areas) [4], which could predict cognitive decline after 2 years of longitudinal follow-up since patients with AD have been demonstrated to show extensive early deposition of neuropathological lesions in these cerebral areas [77]. Therefore, the neurocognitive impairment of the visuospatial working memory in the ADFH individuals with ApoE-4 heterozygotes in the present study is still worth considering in terms of how to maintain or even improve this cognitive function, since they still exhibit comparable levels of AD- and neurocognition-related molecular biomarkers, as shown in the present study.

Actually, it has been well established that physical fitness plays an important role in neurocognitive deficits in individuals with a higher risk of developing AD [48]. In the present study, although the correlations between the event-related neural oscillatory performance and any physical fitness scores did not reach a significant level in either group, cardiorespiratory capability showed significant correlations with the neuropsychological performances in the ApoE-4 group and across both groups. Further, the muscle strength scores (e.g., grip and arm curl) showed significant correlations with ARs across both groups. Since ApoE deficiency is associated with reduced skeletal muscle blood flow and decreased endothelial nitric oxide production [78], physical exercise aimed at improving physical fitness performance has been seen to be reduced in many ApoE-deficiency studies. Etnier et al. (2007) found aerobic fitness (i.e., VO_2max_) to be a positive predictor of neuropsychological performance on memory-related tasks only for ADFH individuals with ApoE-4 homozygotes, but not in the case of the ApoE-4 heterozygotes and ApoE-4 noncarriers [29]. Somewhat inconsistent with the findings of Etnier et al. (2007) [29], in the present study, we found that individuals with ApoE-4 heterozygotes showed significant correlations between the estimated VO_2max_ and neuropsychological performance (i.e., RTs and ARs) when participants performed the visuospatial working memory task. Individuals with higher cardiorespiratory fitness have been reported to exhibit better performance on a wide variety of cognitive tasks [79,80]. In individuals with MCI, a transitional stage from normal aging to dementia, their deficits on the neurophysiological (e.g., event-related P3 potential) index when performing a task-switching paradigm were associated with cardiorespiratory capability, although they had comparable performance on most parts of physical fitness tests as compared to the controls [81]. Through acute and chronic exercise interventions, neurocognitive performance (e.g., ARs, RTs, and event-related potential P3 amplitudes) has been shown to be effectively facilitated after aerobic and resistance exercise in individuals with MCI when performing various cognitive tasks [48,82,83,84]. Most importantly, only a change in cardiorespiratory fitness prior to and after exercise intervention was significantly correlated with changes in the levels of brain-derived neurotrophic factors [48]. Combining the findings of the present and previous studies, cardiorespiratory capability seems to have clinical significance related to neuropsychological and neurophysiological indices in individuals at higher (genetic) risk for developing AD.

The present investigation provides support for neurophysiological mechanisms of visuospatial working memory deficits and an association between cardiorespiratory fitness and ApoE-4 allele in individuals with ADFH. However, there are limitations that must be acknowledged. First, ApoE-4 allele has been reported to associate with lower Aβ1-42 levels in peripheral blood samples [85]. There were only sixteen participants with a single copy of APOE-4 screened from 110 ADFH participants, which could limit the generalizability and reliability of the current biochemical findings (i.e., the lack of group differences). Additionally, the relationship between the ApoE-4 allele and cognitive declines showed a gene–dose effect in elderly men and women [16,17], with homozygotes showing more risk of developing impaired cognitive function than heterozygotes. However, in the present study, although all ADFH participants with ApoE-4 were heterozygotes, they still exhibited worse neurocognitive performance in the higher working-memory-loaded task as compared to the ADFH with non-ApoE-4 carriers. Nevertheless, the presence of two copies of the ApoE-4 allele increases the risk of late-onset AD by about 12 times, and the presence of one copy increases the risk by about 3.7 times. Future research is warranted in this area, possibly examining the interactive effects of aerobic fitness and the ApoE genotype with a larger number of ADFH individuals with homozygous/heterozygous ApoE-4 carriers. Second, inferences about the causal flow between aerobic fitness and the ApoE genotype are not possible given the cross-sectional design since cardiorespiratory fitness and VO_2max_ trainability are associated with genetic factors. This limitation can only be addressed through a longitudinal aerobic-exercise intervention study intended to examine changes in neurocognitive performance relative to ApoE-4 status. However, ApoE polymorphism is a genetic factor contributing to variability in the differences in physical fitness (e.g., cardiorespiratory fitness) responses to chronic exercise training [86,87,88,89]. Future studies using an experimental design should be viewed with caution at this point. Third, the levels of serum Aβ1-40 and Aβ1-42 biomarkers were analyzed in the present study. Along with the development of ultrahigh-sensitivity assay technologies, such as multiplexed flowmetric analysis and single-molecule array, peripheral blood samples might provide an indirect and precise detection of Aβ1-40, Aβ1-42, and tau protein depositions in the brain for prevalent neurodegenerative diseases [90,91,92]. However, previous studies reported that, as compared to the measurement of plasma levels, CSF levels of Aβ1-40 and Aβ1-42 were useful to support the clinical diagnosis of AD, and there were no correlations between plasma and CSF compartments [93,94]. Thus, care must still be taken when generalizing the present findings at this stage.

Expanding the existing research findings in ADFH individuals with ApoE-4 carriers, although such a group has been demonstrated to show subclinical cognitive problems [24,25,26], the ApoE-4 group relative to the non-ApoE-4 group at this stage only partly exhibited worse neuropsychological (e.g., lower ARs) and neurophysiological (greater decreases in oscillatory power at the alpha band) in the higher working-memory-load (i.e., 4-item condition), but not the AD and neurocognition-related molecular biomarkers in the present study. However, ADFH individuals with ApoE-4 carriers are at increased risk of developing AD due to environmental, hereditary, and health risk factors shared with affected parents [95]. Furthermore, individuals at the highest risk for AD are considered to have the smallest cognitive reserves but to show the greatest benefit from cardiorespiratory fitness [29]. In the present study, cardiorespiratory fitness was found to be positively associated with neuropsychological performance in a cognitive task in ADFH individuals at the most genetic risk for AD. The present findings extend current knowledge and suggest that regular physical exercise designed to improve cardiorespiratory capacity may ameliorate visuospatial working-memory declines and further delay the onset of AD in such individuals.

## Figures and Tables

**Table 1:** Demographic characteristics (mean ± SD) of the ApoE-4 and non-APOE-4 groups.

	ApoE-4	Non-ApoE-4	*p*
Age (years)	53.19 ± 8.57	53.94 ± 7.09	0.789
Gender (male/female)	7/9	5/11	0.465
Education (years)	15.06 ± 3.17	14.19 ± 2.43	0.388
Systolic pressure (mmHg)	118.06 ± 16.43	126.13 ± 19.12	0.211
Diastolic pressure (mmHg)	78.63 ± 12.65	81.56 ± 13.20	0.525
Height (cm)	163.41 ± 5.76	159.76 ± 6.95	0.117
Weight (kg)	63.99 ± 12.22	59.05 ± 8.43	0.193
Body Mass Index (kg/m^2^)	23.81 ± 3.17	23.12 ± 2.83	0.525
Social participation	9.88 ± 2.85	10.31 ± 2.98	0.674
MMSE	29.75 ± 0.45	29.50 ± 0.63	0.207
MoCA	28.75 ± 1.29	29.06 ± 0.93	0.438
ACE-III	95.50 ± 4.87	96.19 ± 2.64	0.623
BDI-II	3.75 ± 3.11	1.81 ± 2.54	0.063
Grip (kg)	31.68 ± 9.28	29.82 ± 7.15	0.531
Arm Curl (number)	27.25 ± 10.46	24.56 ± 7.54	0.411
Chair Stand (sec)	17.16 ± 4.84	16.38 ± 4.39	0.882
8-Foot Up-and-Go (sec)	5.14 ± 1.17	4.91 ± 0.57	0.496
Back Scratch (cm)	2.86 ± 5.75	5.54 ± 7.71	0.274
Chair Sit-and-Reach (cm)	1.56 ± 16.00	9.18 ± 10.84	0.128
VO_2max_ (mL/kg/min)	28.00 ± 10.23	31.02 ± 10.12	0.408

MMSE: Mini-Mental State Examination; MoCA: Montreal Cognitive Assessment; ACE-III: Addenbrooke’s Cognitive Examination-III; BDI: Beck depression inventory.

**Table 2:** Biochemical values (mean ± SD) of the ApoE-4 and non-ApoE-4 groups.

	ApoE-4	Non-ApoE-4	*t*	*p*
	*n* = 16	*n* = 16		
IL-1β (pg/mL)	0.05 ± 0.06	0.06 ± 0.10	−0.59	0.561
IL-6 (pg/mL)	0.72 ± 1.13	0.22 ± 0.25	1.74	0.100
IL-8 (pg/mL)	3.21 ± 1.89	2.50 ± 1.78	1.09	0.284
BDNF (ng/mL)	8.47 ± 6.43	10.57 ± 5.00	−1.03	0.311
Aβ1-40 (pg/mL)	57.54 ± 35.40	46.23 ± 32.19	0.95	0.352
Aβ1-42 (pg/dL)	27.01 ± 21.20	25.76 ± 23.66	0.16	0.876

IL: interleukin; BDNF: brain-derived neurotrophic factor; Aβ: amyloid-β.

**Table 3:** The relationships between senior functional physical fitness/cardiorespiratory tests and neuropsychological performance in the visuospatial working memory paradigm in the ApoE-4 and non-ApoE-4 groups and all participants.

Group		Grip	Arm Curl	Chair Stand	8-Foot Up-and-Go	Back Scratch	Chair Sit-and-Reach	VO_2max_
ApoE-4								
	2-item AR	*r* = 0.48, *p* = 0.059	*r* = 0.38, *p* = 0.142	*r* = 0.25, *p* = 0.351	*r* = 0.29, *p* = 0.273	*r* = −0.34, *p* = 0.902	*r* = 0.15, *p* = 0.590	*r* = 0.59, *p* = 0.017
	4-item AR	*r* = 0.46, *p* = 0.071	*r* = 0.41, *p* = 0.119	*r* = 0.24, *p* = 0.366	*r* = 0.43, *p* = 0.100	*r* = −0.45, *p* = 0.077	*r* = −0.28, *p* = 0.295	*r* = 0.63, *p* = 0.009
	2-item RT	*r* = −0.41, *p* = 0.120	*r* = −0.18, *p* = 0.518	*r* = −0.08, *p* = 0.779	*r* = −0.40, *p* = 0.129	*r* = 0.35, *p* = 0.179	*r* = 0.07, *p* = 0.805	*r* = −0.52, *p* = 0.039
	4-item RT	*r* = −0.30, *p* = 0.254	*r* = −0.06, *p* = 0.826	*r* = 0.07, *p* = 0.792	*r* = −0.34, *p* = 0.204	*r* = 0.56, *p* = 0.023	*r* = 0.26, *p* = 0.328	*r* = −0.55, *p* = 0.027
non-ApoE-4								
	2-item AR	*r* = 0.32, *p* = 0.230	*r* = 0.40, *p* = 0.121	*r* = 0.46, *p* = 0.074	*r* = −0.39, *p* = 0.131	*r* = −0.12, *p* = 0.672	*r* = 0.51, *p* = 0.042	*r* = 0.56, *p* = 0.024
	4-item AR	*r* = 0.44, *p* = 0.090	*r* = 0.22, *p* = 0.410	*r* = 0.19, *p* = 0.488	*r* = −0.34, *p* = 0.202	*r* = −0.10, *p* = 0.726	*r* = 0.38, *p* = 0.149	*r* = 0.46, *p* = 0.072
	2-item RT	*r* = −0.24, *p* = 0.377	*r* = −0.37, *p* = 0.160	*r* = −0.10, *p* = 0.707	*r* = 0.17, *p* = 0.538	*r* = −0.23, *p* = 0.399	*r* = −0.49, *p* = 0.056	*r* = −0.31, *p* = 0.238
	4-item RT	*r* = −0.11, *p* = 0.687	*r* = −0.19, *p* = 0.478	*r* = −0.46, *p* = 0.865	*r* = 0.25, *p* = 0.352	*r* = −0.19, *p* = 0.489	*r* = −0.29, *p* = 0.269	*r* = −0.24, *p* = 0.366
All participants								
	2-item AR	*r* = 0.37, *p* = 0.038	*r* = 0.37, *p* = 0.035	*r* = 0.32, *p* = 0.078	*r* = −0.00, *p* = 0.994	*r* = −0.12, *p* = 0.527	*r* = 0.25, *p* = 0.160	*r* = 0.52, *p* = 0.003
	4-item AR	*r* = 0.37, *p* = 0.035	*r* = 0.26, *p* = 0.147	*r* = 0.20, *p* = 0.273	*r* = 0.18, *p* = 0.323	*r* = −0.17, *p* = 0.349	*r* = 0.03, *p* = 0.876	*r* = 0.56, *p* = 0.001
	2-item RT	*r* = −0.30, *p* = 0.092	*r* = −0.25, *p* = 0.174	*r* = −0.08, *p* = 0.660	*r* = −0.15, *p* = 0.399	*r* = −0.02, *p* = 0.903	*r* = −0.19, *p* = 0.306	*r* = −0.40, *p* = 0.022
	4-item RT	*r* = −0.19, *p* = 0.310	*r* = −0.07, *p* = 0.690	*r* = 0.03, *p* = 0.859	*r* = −0.11, *p* = 0.556	*r* = 0.09, *p* = 0.638	*r* = −0.03, *p* = 0.893	*r* = −0.42, *p* = 0.017

## References

[1] Bastiaansen M.C., Posthuma D., Groot P.F., de Geus E.J. (2002). Event-related alpha and theta responses in a visuo-spatial working memory task. Clin. Neurophysiol..

[2] Proskovec A.L., Heinrichs-Graham E., Wilson T.W. (2019). Load modulates the alpha and beta oscillatory dynamics serving verbal working memory. Neuroimage.

[3] Jost B.C., Grossberg G.T. (1995). The natural history of Alzheimer’s disease: A brain bank study. J. Am. Geriatr. Soc..

[4] Small G.W., Ercoli L.M., Silverman D.H., Huang S.C., Komo S., Bookheimer S.Y., Lavretsky H., Miller K., Siddarth P., Rasgon N.L. (2000). Cerebral metabolic and cognitive decline in persons at genetic risk for Alzheimer’s disease. Proc. Natl. Acad. Sci. USA.

[5] Corder E.H., Saunders A.M., Strittmatter W.J., Schmechel D.E., Gaskell P.C., Small G.W., Roses A.D., Haines J.L., Pericak-Vance M.A. (1993). Gene dose of apolipoprotein E type 4 allele and the risk of Alzheimer’s disease in late onset families. Science.

[6] Corder E.H., Saunders A.M., Risch N.J., Strittmatter W.J., Schmechel D.E., Gaskell P.C., Rimmler J.B., Locke P.A., Conneally P.M., Schmader K.E. (1994). Protective effect of apolipoprotein E type 2 allele for late onset Alzheimer disease. Nat. Genet..

[7] Reiman E.M., Chen K., Liu X., Bandy D., Yu M., Lee W., Ayutyanont N., Keppler J., Reeder S.A., Langbaum J.B. (2009). Fibrillar amyloid-beta burden in cognitively normal people at 3 levels of genetic risk for Alzheimer’s disease. Proc. Natl. Acad. Sci. USA.

[8] Chen Y., Durakoglugil M.S., Xian X., Herz J. (2010). ApoE4 reduces glutamate receptor function and synaptic plasticity by selectively impairing ApoE receptor recycling. Proc. Natl. Acad. Sci. USA.

[9] Kim J., Yoon H., Basak J., Kim J. (2014). Apolipoprotein E in synaptic plasticity and Alzheimer’s disease: Potential cellular and molecular mechanisms. Mol. Cells.

[10] Nwabuisi-Heath E., Rebeck G.W., Ladu M.J., Yu C. (2014). ApoE4 delays dendritic spine formation during neuron development and accelerates loss of mature spines in vitro. ASN Neuro.

[11] Dumanis S.B., DiBattista A.M., Miessau M., Moussa C.E., Rebeck G.W. (2013). APOE genotype affects the pre-synaptic compartment of glutamatergic nerve terminals. J. Neurochem..

[12] Bookheimer S.Y., Strojwas M.H., Cohen M.S., Saunders A.M., Pericak-Vance M.A., Mazziotta J.C., Small G.W. (2000). Patterns of brain activation in people at risk for Alzheimer’s disease. N. Engl. J. Med..

[13] Reiman E.M., Caselli R.J., Yun L.S., Chen K., Bandy D., Minoshima S., Thibodeau S.N., Osborne D. (1996). Preclinical evidence of Alzheimer’s disease in persons homozygous for the epsilon 4 allele for apolipoprotein E. N. Engl. J. Med..

[14] Wishart H.A., Saykin A.J., McAllister T.W., Rabin L.A., McDonald B.C., Flashman L.A., Roth R.M., Mamourian A.C., Tsongalis G.J., Rhodes C.H. (2006). Regional brain atrophy in cognitively intact adults with a single APOE epsilon4 allele. Neurology.

[15] Persson J., Lind J., Larsson A., Ingvar M., Cruts M., Van Broeckhoven C., Adolfsson R., Nilsson L.G., Nyberg L. (2006). Altered brain white matter integrity in healthy carriers of the APOE epsilon4 allele: A risk for AD?. Neurology.

[16] Feskens E.J., Havekes L.M., Kalmijn S., de Knijff P., Launer L.J., Kromhout D. (1994). Apolipoprotein e4 allele and cognitive decline in elderly men. Br. Med. J..

[17] Yaffe K., Cauley J., Sands L., Browner W. (1997). Apolipoprotein E phenotype and cognitive decline in a prospective study of elderly community women. Arch. Neurol..

[18] Petersen R.C., Smith G.E., Ivnik R.J., Tangalos E.G., Schaid D.J., Thibodeau S.N., Kokmen E., Waring S.C., Kurland L.T. (1995). Apolipoprotein E status as a predictor of the development of Alzheimer’s disease in memory-impaired individuals. JAMA.

[19] Van Duijn C.M., Hofman A. (1992). Risk factors for Alzheimer’s disease: The EURODEM collaborative re-analysis of case-control studies. Neuroepidemiology.

[20] Bendlin B.B., Ries M.L., Canu E., Sodhi A., Lazar M., Alexander A.L., Carlsson C.M., Sager M.A., Asthana S., Johnson S.C. (2010). White matter is altered with parental family history of Alzheimer’s disease. Alzheimers Dement..

[21] La Rue A., Matsuyama S.S., McPherson S., Sherman J., Jarvik L.F. (1992). Cognitive performance in relatives of patients with probable Alzheimer disease: An age at onset effect?. J. Clin. Exp. Neuropsychol..

[22] La Rue A., O’Hara R., Matsuyama S.S., Jarvik L.F. (1995). Cognitive changes in young-old adults: Effect offamily histoηr of dementia. J. Clin. Exp. Neuropsychol..

[23] Caselli R.J., Reiman E.M., Osborne D., Hentz J.G., Baxter L.C., Hernandez J.L., Alexander G.G. (2004). Longitudinal changes in cognition and behavior in asymptomatic carriers of the APOE e4 allele. Neurology.

[24] Greenwood P.M., Lambert C., Sunderland T., Parasuraman R. (2005). Effects of apolipoprotein E genotype on spatial attention, working memory, and their interaction in healthy middle-aged adults: Results from the National Institute of Mental Health’s BIOCARD study. Neuropsychology.

[25] Levy J.A., Bergeson J., Putnam K., Rosen V., Cohen R., Lalonde F., Mirza N., Linker G., Sunderland T. (2004). Context-specific memory and apolipoprotein E (APOE) epsilon 4: Cognitive evidence from the NIMH prospective study of risk for Alzheimer’s disease. J. Int. Neuropsychol. Soc..

[26] Rosen V.M., Bergeson J.L., Putnam K., Harwell A., Sunderland T. (2002). Working memory and apolipoprotein E: What’s the connection?. Neuropsychologia.

[27] Laczó J., Andel R., Vlček K., Macoška V., Vyhnálek M., Tolar M., Bojar M., Hort J. (2011). Spatial navigation and APOE in amnestic mild cognitive impairment. Neurodegener. Dis..

[28] Head D., Bugg J.M., Goate A.M., Fagan A.M., Mintun M.A., Benzinger T., Holtzman D.M., Morris J.C. (2012). Exercise engagement as a moderator of the effects of APOE genotype on amyloid deposition. Arch. Neurol..

[29] Etnier J.L., Caselli R.J., Reiman E.M., Alexander G.E., Sibley B.A., Tessier D., McLemore E.C. (2007). Cognitive performance in older women relative to ApoE-epsilon4 genotype and aerobic fitness. Med. Sci. Sports Exerc..

[30] Jack C.R., Bennett D.A., Blennow K., Carrillo M.C., Dunn B., Haeberlein S.B., Holtzman D.M., Jagust W., Jessen F., Karlawish J. (2018). NIA-AA Research Framework: Toward a biological definition of Alzheimer’s disease. Alzheimers Dement..

[31] Morris J.C. (1997). Clinical assessment of Alzheimer’s disease. Neurology.

[32] Selkoe D.J. (2002). Alzheimer’s disease is a synaptic failure. Science.

[33] Golde T.E., Dickson D., Hutton M. (2006). Filling the gaps in the abeta cascade hypothesis of Alzheimer’s disease. Curr. Alzheimer Res..

[34] Karran E., Mercken M., De Strooper B. (2011). The amyloid cascade hypothesis for Alzheimer’s disease: An appraisal for the development of therapeutics. Nat. Rev. Drug Discov..

[35] Castellano J.M., Castellano J.M., Kim J., Stewart F.R., Jiang H., DeMattos R.B., Patterson B.W., Fagan A.M., Morris J.C., Mawuenyega K.G. (2011). Human apoE isoforms differentially regulate brain amyloid-β peptide clearance. Sci. Transl. Med..

[36] Mosconi L., De Santi S., Brys M., Tsui W.H., Pirraglia E., Glodzik-Sobanska L., Rich K.E., Switalski R., Mehta P.D., Pratico D. (2008). Hypometabolism and altered cerebrospinal fluid markers in normal apolipoprotein E E4 carriers with subjective memory complaints. Biol. Psychiatry.

[37] Galluzzi S., Marizzoni M., Babiloni C., Albani D., Antelmi L., Bagnoli C., Bartres-Faz D., Cordone S., Didic M., Farotti L. (2016). Clinical and biomarker profiling of prodromal Alzheimer’s disease in workpackage 5 of the Innovative Medicines Initiative PharmaCog project: A ‘European ADNI study’. J. Intern. Med..

[38] Diniz B.S., Teixeira A.L., Ojopi E.B., Talib L.L., Mendonça V.A., Gattaz W.F., Forlenza O.V. (2010). Higher serum sTNFR1 level predicts conversion from mild cognitive impairment to Alzheimer’s disease. J. Alzheimers Dis..

[39] McGeer E.G., McGeer P.L. (2010). Neuroinflammation in Alzheimer’s disease and mild cognitive impairment: A field in its infancy. J. Alzheimers Dis..

[40] Forlenza O.V., Diniz B.S., Talib L.L., Mendonca V.A., Ojopi E.B., Gattaz W.F., Teixeira A.L. (2009). Increased serum IL-1beta level in Alzheimer’s disease and mild cognitive impairment. Dement. Geriatr. Cogn. Disord..

[41] Guerreiro R.J., Santana I., Bras J.M., Santiago B., Paiva A., Oliveira C. (2007). Peripheral inflammatory cytokines as biomarkers in Alzheimer’s disease and mild cognitive impairment. Neurodegener. Dis..

[42] Gezen-Ak D., Dursun E., Hanağası H., Bilgiç B., Lohman E., Araz Ö.S., Atasoy I.L., Alaylıoğlu M., Önal B., Gürvit H. (2013). BDNF, TNFα, HSP90, CFH, and IL-10 serum levels in patients with early or late onset Alzheimer’s disease or mild cognitive impairment. J. Alzheimers Dis..

[43] Peng S., Wuu J., Mufson E.J., Fahnestock M. (2005). Precursor form of brain-derived neurotrophic factor and mature brain-derived neurotrophic factor are decreased in the pre-clinical stages of Alzheimer’s disease. J. Neurochem..

[44] Alvarez A., Aleixandre M., Linares C., Masliah E., Moessler H. (2014). Apathy and APOE4 are associated with Reduced BDNF Levels in Alzheimer’s disease. J. Alzheimers Dis..

[45] Adlard P.A., Perreau V.M., Pop V., Cotman C.W. (2005). Voluntary exercise decreases amyloid load in a Transgenic model of Alzheimer’s disease. J. Neurosci..

[46] Erickson K.I., Voss M.W., Prakash R.S., Basak C., Szabo A., Chaddock L., Kim J.S., Heo S., Alves H., White S.M. (2011). Exercise training increases size of hippocampus and improves memory. Proc. Natl. Acad. Sci. USA.

[47] Nyberg J., Åberg M.A., Schiöler L., Nilsson M., Wallin A., Torén K., Kuhn H.G. (2014). Cardiovascular and cognitive fitness at age 18 and risk of early-onset dementia. Brain.

[48] Tsai C.L., Pai M.C., Ukropec J., Ukropcová B. (2019). Distinctive effects of aerobic and resistance exercise modes on neurocognitive and biochemical changes in individuals with mild cognitive impairment. Curr. Alzheimer Res..

[49] Ward L.M. (2003). Synchronous neural oscillations and cognitive processes. Trends Cogn. Sci..

[50] Basar E., Schurmann M., Demiralp T., Basar-Eroglu C., Ademoglu A. (2001). Event-related oscillations are ‘real brain responses’-Wavelet analysis and new strategies. Int. J. Psychophysiol..

[51] Klimesch W. (2012). Alpha-band oscillations, attention, and controlled access to stored information. Trends Cogn. Sci..

[52] Rispoli V., Ragusa S., Nisticò R., Marra R., Russo E., Leo A., Felicitá V., Rotiroti D. (2013). Huperzine a restores cortico-hippocampal functional connectivity after bilateral AMPA lesion of the nucleus basalis of meynert. J. Alzheimers Dis..

[53] Wianda E., Ross B. (2019). The roles of alpha oscillation in working memory retention. Brain Behav..

[54] Cohen M.X. (2014). Analyzing Neural Time Series Data: Theory and Practice.

[55] Rikli R.E., Jones C.J. (2012). Senior Fitness Test Manual.

[56] Kline G.M., Porcari J.P., Hintermeister R., Freedson P.S., Ward A., McCarron R.F., Ross J., Rippe J.M. (1987). Estimation of VO2max from a one-mile track walk, gender, age, and body weight. Med. Sci. Sports Exerc..

[57] Parra M.A., Abrahams S., Logie R.H., Méndez L.G., Lopera F., Della Sala S. (2010). Visual short-term memory binding deficits in familial Alzheimer’s disease. Brain.

[58] McCarthy G., Puce A., Constable R.T., Krystal J.H., Gore J.C., Goldman-Rakic P. (1996). Activation of human prefrontal cortex during spatial and nonspatial working memory tasks measured by functional MRI. Cereb. Cortex.

[59] Knott V., Millar A., Dulude L., Bradford L., Alwahhabi F., Lau T., Shea C., Wiens A. (2004). Event-related potentials in young and elderly adults during a visual spatial working memory task. Clin. EEG Neurosci..

[60] Hsu T.Y., Tseng P., Liang W.K., Cheng S.K., Juan C.H. (2014). Transcrainal direct current stimulation over right posterior parietal cortex changes prestimulus alpha oscillation in viusal short-term memory task. Neuroimage.

[61] Wang C.H., Lo Y.H., Pan C.Y., Chen F.C., Liang W.K., Tsai C.L. (2015). Frontal midline theta as a neurophysiological correlate for deficits of attentional orienting in children with developmental coordination disorder. Psychophysiology.

[62] Wang C.H., Tseng Y.T., Liu D., Tsai C.L. (2017). Neural oscillation reveals deficits in visuospatial working memory in children with developmental coordination disorder. Child Dev..

[63] Roach B.J., Mathalon D.H. (2008). Event-related EEG time-frequency analysis: An overview of measures and an analysis of early gamma band phase locking in schizophrenia. Schizophr. Bull..

[64] Chu C.H., Yang K.T., Song T.F., Liu J.H., Hung T.M., Chang Y.K. (2016). Cardiorespiratory fitness is associated with executive control in late-middle-aged adults: An event-related (De) synchronization (ERD/ERS) study. Front. Psychol..

[65] Benjamini Y., Yekutieli D. (2001). The control of the false discovery rate in multiple testing under dependency. Ann. Stat..

[66] Cohen J. (1973). Eta-squared and partial eta-squared in fixed factor ANOVA designs. Educ. Psychol. Meas..

[67] Klimesch W., Schack B., Sauseng P. (2005). The functional significance of theta and upper alpha oscillations. Exp. Psychol..

[68] Sauseng P., Klimesch W., Schabus M., Doppelmayr M. (2005). Fronto-parietal EEG coherence in theta and upper alpha reflect central executive functions of working memory. Int. J. Psychophysiol..

[69] Klimesch W., Doppelmayr M., Russegger H., Pachinger T., Schwaiger J. (1998). Induced alpha band power changes in the human EEG and attention. Neurosci. Lett..

[70] Stipacek A., Grabner R.H., Neuper C., Fink A., Neubauer A.C. (2003). Sensitivity of human EEG alpha band desynchronization to different working memory components and increasinglevels of memory load. Neurosci. Lett..

[71] Green J., Levey A.I. (1999). Event-related potential changes in groups at increased risk for Alzheimer disease. Arch. Neurol..

[72] Sun X., Chiu C.C., Liebson E., Crivello N.A., Wang L., Claunch J., Folstein M., Rosenberg I., Mwamburi D.M., Peter I. (2009). Depression and plasma amyloid beta peptides in the elderly with and without the apolipoprotein E4 allele. Alzheimer Dis. Assoc. Disord..

[73] Babiloni C., Benussi L., Binetti G., Cassetta E., Dal Forno G., Del Percio C., Ferreri F., Ferri R., Frisoni G., Ghidoni R. (2006). Apolipoprotein E and alpha brain rhythms in mild cognitive impairment: A multicentric electroencephalogram study. Ann. Neurol..

[74] Baddeley A.D. (1996). Exploring the central executive. Q. J. Exp. Psychol..

[75] Cantero J.L., Atienza M., Cruz-Vadell A., Suarez-Gonzalez A., Gil-Neciga E. (2009). Increased synchronization and decreased neural complexity underlie thalamocortical oscillatory dynamics in mild cognitive impairment. Neuroimage.

[76] Bookheimer S., Burggren A. (2009). APOE-4 genotype and neurophysiological vulnerability to Alzheimer’s and cognitive aging. Annu. Rev. Clin. Psychol..

[77] Price J.L., Morris J.C. (1999). Tangles and plaques in nondemented aging and “preclinical” Alzheimer’s disease. Ann. Neurol..

[78] Niebauer J., Maxwell A.J., Lin P.S., Tsao P.S., Kosek J., Bernstein D., Cooke J.P. (1999). Impaired aerobic capacity in hypercholesterolemic mice: Partial reversal by exercise training. Am. J. Physiol..

[79] Themanson J.R., Hillman C.H., Curtin J.J. (2006). Age and physical activity influences on action monitoring during task switching. Neurobiol. Aging.

[80] Tsai C.L., Pan C.Y., Chen F.C., Tseng Y.T. (2017). Open- and closed-skill exercise interventions produce different neurocognitive effects on executive functions in the elderly: A 6-month randomized, controlled trial. Front. Aging Neurosci..

[81] Tsai C.L., Pai M.C., Ukropec J., Ukropcová B. (2016). The role of physical fitness in the neurocognitive performance of task switching in older persons with mild cognitive impairment. J. Alzheimers Dis..

[82] Baker L.D., Frank L.L., Foster-Schubert K., Green P.S., Wilkinson C.W., McTiernan A., Plymate S.R., Fishel M.A., Watson G.S., Cholerton B.A. (2010). Effects of aerobic exercise on mild cognitive impairment: A controlled trial. Arch. Neurol..

[83] Ten Brinke L.F., Bolandzadeh N., Nagamatsu L.S., Hsu C.L., Davis J.C., Miran-Khan K., Liu-Ambrose T. (2015). Aerobic exercise increases hippocampal volume in older women with probable mild cognitive impairment: A 6-month randomised controlled trial. Br. J. Sports Med..

[84] Tsai C.L., Ukropec J., Ukropcová B., Pai M.C. (2018). An acute bout of aerobic or strength exercise specifically modifies circulating exerkine levels and neurocognitive functions in elderly individuals with mild cognitive impairment. NeuroImage Clin..

[85] Metti A.L., Cauley J.A., Ayonayon H.N., Harris T.B., Rosano C., Williamson J.D., Yaffe K. (2013). The demographic and medical correlates of plasma aβ40 and aβ42. Alzheimer Dis. Assoc. Disord..

[86] Hagberg J.M., Ferrell R.E., Katzel L.I., Dengel D.R., Sorkin J.D., Goldberg A.P. (1999). Apolipoprotein E genotype and exercise training induced increases in high-density lipoprotein (HDL)- and HDL2-cholesterol levels in overweight men. Metabolism.

[87] Leon A.S., Togashi K., Rankinen T., Després J.P., Rao D.C., Skinner J.S., Wilmore J.H., Bouchard C. (2004). Association of apolipoprotein E polymorphism with blood lipids and maximal oxygen uptake in the sedentary state and after exercise training in the HERITAGE family study. Metabolism.

[88] Meckes C.L., Moytna N.A., Tsongalis G., Miles M. (2001). The increase in maximal oxygen uptake with exercise training is reduced in subjects homozygous for the apolipoprotein E3 allele. Circulation.

[89] Thompson P.D., Tsongalis G.J., Seip R.L., Bilbie C., Miles M., Zoeller R., Visich P., Gordon P., Angelopoulos T.J., Pescatello L. (2004). Apolipoprotein E genotype and changes in serum lipids and maximal oxygen uptake with exercise training. Metabolism.

[90] Lue L.F., Sabbagh M.N., Chiu M.J., Jing N., Snyder N.L., Schmitz C., Guerra A., Belden C.M., Chen T.F., Yang C.C. (2017). Plasma levels of Aβ42 and tau identified probable Alzheimer’s dementia: Findings in two cohorts. Front. Aging Neurosci..

[91] Tzen K.Y., Yang S.Y., Chen T.F., Cheng T.W., Horng H.E., Wen H.P., Huang Y.Y., Shiue C.Y., Chiu M.J. (2014). Plasma Aβ but not tau is related to brain PiB retention in early Alzheimer’s disease. ACS Chem. Neurosci..

[92] Yang S.Y., Chiu M.J., Chen T.F., Lin C.H., Jeng J.S., Tang S.C., Lee Y.F., Yang C.C., Liu B.H., Chen H.H. (2017). Analytical performance of reagent for assaying tau protein in human plasma and feasibility study screening neurodegenerative diseases. Sci. Rep..

[93] Mehta P.D., Pirttilä T., Mehta S.P., Sersen E.A., Aisen P.S., Wisniewski H.M. (2000). Plasma and cerebrospinal fluid levels of amyloid beta proteins 1-40 and 1-42 in Alzheimer disease. Arch. Neurol..

[94] Vanderstichele H., Van Kerschaver E., Hesse C., Davidsson P., Buyse M.A., Andreasen N., Minthon L., Wallin A., Blennow K., Vanmechelen E. (2000). Standardization of measurement of beta-amyloid(1-42) in cerebrospinal fluid and plasma. Amyloid.

[95] Sager M.A., Hermann B., La Rue A. (2005). Middle-aged children of persons with Alzheimer’s disease: APOE genotypes and cognitive function in the Wisconsin Registry for Alzheimer’s Prevention. J. Geriatr. Psychiatry Neurol..

